# New differentially expressed genes and differential DNA methylation underlying refractory epilepsy

**DOI:** 10.18632/oncotarget.13642

**Published:** 2016-11-26

**Authors:** Xi Liu, Shu Ou, Tao Xu, Shiyong Liu, Jinxian Yuan, Hao Huang, Lu Qin, Hui Yang, Lifen Chen, Xinjie Tan, Yangmei Chen

**Affiliations:** ^1^ Department of Neurology, The Second Affiliated Hospital of Chongqing Medical University, Yuzhong District, Chongqing, 400010, China; ^2^ Epilepsy Center of PLA, Department of Neurosurgery, Xinqiao Hospital, The Third Military Medical University, Shapingba District, Chongqing, 400037, China; ^3^ Department of Neurology, Affiliated Hospital of Zunyi Medical College, Zunyi, 563003, China

**Keywords:** refractory epilepsy, human, epigenetics, DNA methylation, gene expression

## Abstract

Epigenetics underlying refractory epilepsy is poorly understood, especially in patients without distinctive genetic alterations. DNA methylation may affect gene expression in epilepsy without affecting DNA sequences. Herein, we analyzed genome-wide DNA methylation and gene expression in brain tissues of 10 patients with refractory epilepsy using methylated DNA immunoprecipitation linked with sequencing and mRNA Sequencing. Diverse distribution of differentially methylated genes was found in X chromosome, while differentially methylated genes appeared rarely in Y chromosome. 62 differentially expressed genes, such as MMP19, AZGP1, DES, and LGR6 were correlated with refractory epilepsy for the first time. Although general trends of differentially enriched gene ontology terms and Kyoto Encyclopedia of Genes and Genome pathways in this study are consistent with previous researches, differences also exist in many specific gene ontology terms and Kyoto Encyclopedia of Genes and Genome pathways. These findings provide a new genome-wide profiling of DNA methylation and gene expression in brain tissues of patients with refractory epilepsy, which may provide a basis for further study on the etiology and mechanisms of refractory epilepsy.

## INTRODUCTION

65 millions of people were affected by epilepsy in the world according to International League Against Epilepsy (ILAE) [1], and approximately 36% of epilepsy patients were drug-resistant [2]. Varieties of genes encoding channels, receptors, transporters, synaptic transmission, etc. have been associated with different types of epilepsy, among which, some were associated with refractory epilepsy [3, 4, 5, 6]. Many environmental factors, such as economic situation, diet, trauma, stroke, etc. were also associated with epilepsy or seizure [7, 8, 9, 10].

Epigenetic modifications, including DNA methylation, histone modification and aberrant microRNA expression, can affect genomic reprogramming, tissue-specific gene expression and global gene silencing without affecting DNA sequence itself [11, 12]. The most common form of DNA methylation occurs at the 5’carbon of cytosine in CpG dinucleotides which often locates in CpG islands within the promoters [13]. More recently, DNA methylation is raised as one of the main epigenetic mechanisms in epilepsy [14]. Previous genome-wide DNA methylation profiling in epileptic animal models presented altered DNA methylation in promoters of genes, and identified many genes that were associated with epilepsy [15].

DNA methylation in promoter may decrease gene expression, for example, increased methylation of reelin promoter resulted in the decrease of reelin expression in epilepsy model [16]. Katja Kobow and colleagues found a genome-wide distinctive DNA methylation pattern in rat models of pilocarpine-induced epilepsy in addition to an inverse relationship between gene expression and DNA methylation in promoter, exon and intron [17]. Meanwhile, ketogenic diet could attenuate seizure progression via ameliorating DNA methylation [17]. Selective changes in genome-wide DNA methylation and increased DNA-methyltransferase were also discovered in patients with temporal lobe epilepsy (TLE) [18, 19]. However, the profiling of genome-wide DNA methylation and gene expression in patients with refractory epilepsy remains unclear.

In this study, methylated DNA immunoprecipitation linked with sequencing (MeDIP-Seq) and mRNA sequencing (mRNA-Seq) were used to analyze the pattern of genome-wide DNA methylation and gene expression, as well as the relationship between DNA methylation and gene expression. Our findings identified a new distribution pattern of DNA methylation and gene expression in refractory epilepsy patients. Most of the differentially methylated genes (DMG) were methylated in gene element of coding sequences (CDS) and introns. More importantly, some new refractory epilepsy-related genes that have not been documented previously were found in this study.

## RESULTS

### Demographic and clinical characteristics of subjects

The mean age (mean±SD) of the 10 epileptic samples (5 male/5 female) was 17.10±5.84, and the age of epilepsy onset was 6.49±6.16. The mean age of the 10 controls (7 male/3 female) was 39.00±17.40. The detailed data of demographic and clinical characteristics of epileptic samples were presented in Table 1 & Supplementary Table S1.

**Table 1:** Clinical characteristics of patients and controls

Characteristics	Patients	Controls
Age (year)	17.10±5.84	39.00±17.40
Age of onset (year)	6.49±6.16	N/A
Sex (male/female)	5/5	7/3
Frequency of seizures per month		N/A
<10	5	
10∼100	1	
>100	4	
Pattern of seizures		N/A
GS	4	
GTCS+CPS	4	
PS	2	
AEDs before operation		N/A
VPA	9	
CMZ	6	
TPM	3	
PB	2	
OMZ	2	
LEV	5	
LTG	2	
PHT	1	
CZP	1	

GS: generalized seizure; GTCS: generalized tonic clonic seizure; CPS: complex partial seizure; PS: partial seizure; VPA: valproic acid; CMZ: carbamazipine; TPM: topiramate; PB: phenobarbital; OMZ: oxcarbazepine; LEV: levetiracetam; LTG: lamotrigine; PHT: phenytoin; CZP: clonazepam; N/A: non applicable.

### No significant difference in distribution of DNA methylation reads

In each sample, 81632654 methylation reads (49 bp) were sequenced. In epileptic samples, an average 71.20% of the reads were uniquely mapped to the reference genome, and in controls, 70.77% of the reads were uniquely mapped. There was no significant difference of uniquely mapped reads between the two groups by T test (Supplementary Table S2).

Moreover, no significant difference was identified between epileptic samples and controls in 1) genome coverage distribution across sequencing depth, 2) distribution of CpG, CHG, and CHH sites across sequencing depth, 3) reads distribution in genome regions with different CpG density, 4) distribution of reads in different gene elements and repetitive elements, 5) distribution of reads around CpG island and gene body.

### No significant difference in distribution of DNA methylation peaks

In each epileptic sample, an average of 115128.10±21674.80 peaks were identified, covering an average of 152796189.80±26659961.36 bp and 4.87±0.85% of human genome. In controls, the mean identified peaks were 111020.20±25956.50, covering 148106055.60±25489074.79 bp and 4.72±0.81% of human genome (Supplementary Table S2). No significant difference between the two groups was found. In addition, no significant difference in the number of peaks with different length, the distribution of peaks with different CpG density and distribution of peaks in gene elements (including peak number and peak coverage) was observed between epileptic samples and control.

### Analysis of differentially methylated regions (DMR) and DMGs

**Figure 1:**
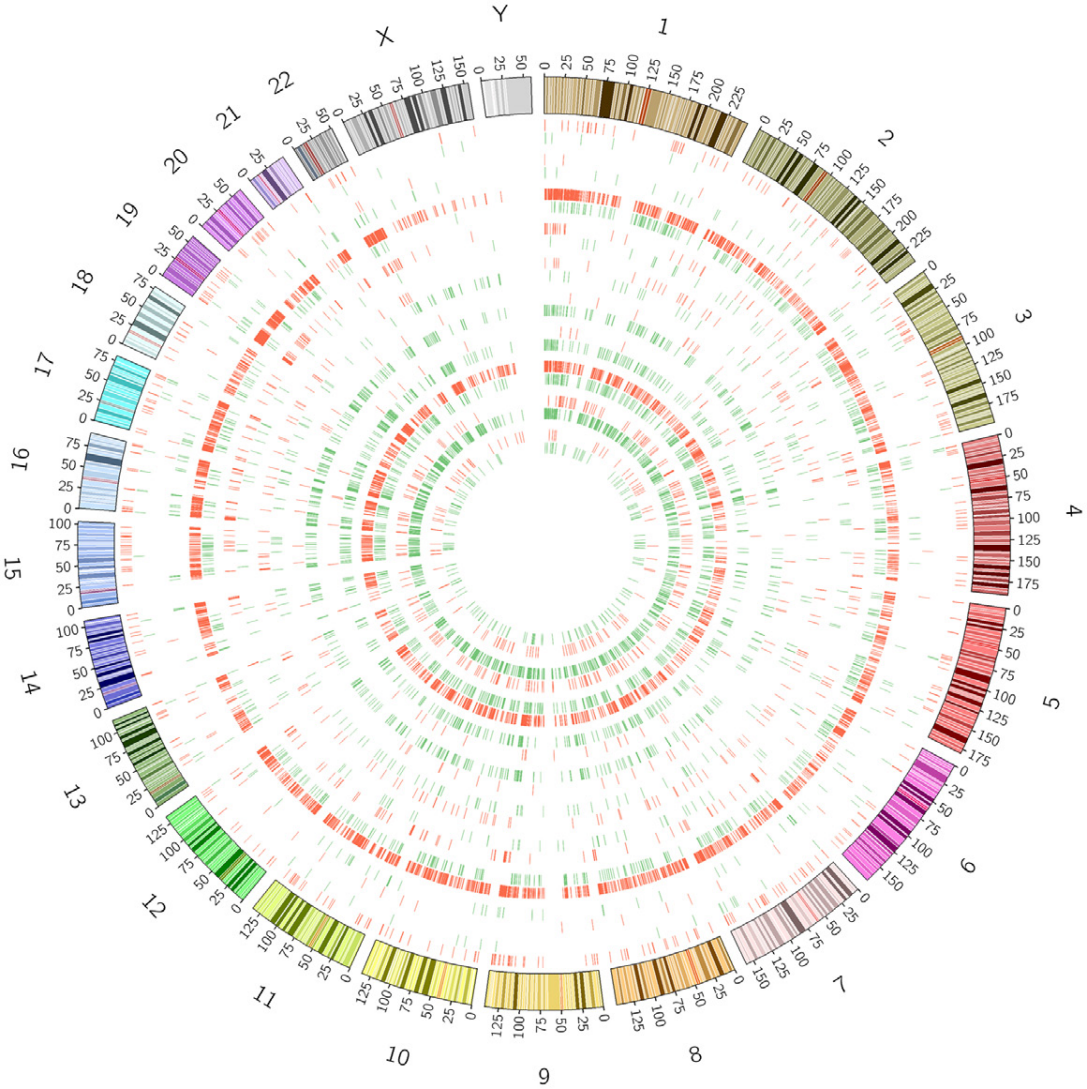
Distribution of differentially methylated genes (DMGs) across genome Hyper-methylated (purple) and hypo-methylated (green) regions in epileptic samples vs. controls were targeted to each chromosome. Diverse distribution of DMGs was found in X chromosome, while DMGs appeared rarely in Y chromosome.

The median of DMRs identified by pairwise comparison was 7604.50, covering a median of 8580791.00 bp. The distribution of DMGs in paired samples was mapped to human genome using Circos [20] (Figure 1). DMGs appeared on all of chromosomes extensively except for Y chromosome in refractory epilepsy patients.

The significant enriched gene ontology (GO) terms of DMGs were presented in Table 2. Most of the DMGs were differentially methylated in gene element of CDS and introns. Significant enrichment of DMGs was observed in GO terms of binding of various molecules, such as ATP binding, ion binding, cation binding, and nucleoside binding. In addition, DMG enrichment was also identified in the GO terms involved in receptor activity, transporter activity, kinase activity, transducer activity and channel activity. Those DMGs participate mainly in the biological processes of adhesion and ion transport.

**Table 2:** GO enrichment analysis of differentially methylated genes

Element	Category	Terms
CDS	C	apical part of cell; apical plasma membrane; axonemal dynein complex; basement membrane; cell surface; cytoplasm; cytoskeleton; cytoskeleton; dynein complex; extracellular matrix; extracellular matrix part;extracellular region; extracellular region part; extracellular space; insoluble fraction; integral to plasma membrane; intracellular; intracellular organelle; intracellular organelle part;intracellular part; intrinsic to plasma membrane; membrane; membrane attack complex; membrane fraction; membrane part; membrane-bounded organelle; myosin complex; myosin filament;nucleolus; organelle; organelle part; plasma membrane; plasma membrane part; platelet alpha granule lumen; proteinaceous extracellular matrix; striated muscle thick filament
	F	GTPase binding; adenyl nucleotide binding; adenyl ribonucleotide binding; ATP binding; calcium ion binding;calmodulin binding; collagen binding; extracellular matrix structural constituent; GTPase regulator activity; guanyl-nucleotide exchange factor activity; microtubule motor activity;motor activity; NAD(P)H oxidase activity; nucleoside binding; phosphotransferase activity, alcohol group as acceptor; purine nucleoside binding; purine nucleotide binding;Ras guanyl-nucleotide exchange factor activity; Rho guanyl-nucleotide exchange factor activity; small GTPase binding
	P	cell adhesion; biological adhesion
Intron	C	synapse; actin cytoskeleton; basement membrane; basolateral plasma membrane; cell; cell junction; cell part;cell projection; cytoskeleton; extracellular matrix; extracellular region part; integral to membrane;integral to plasma membrane; intrinsic to membrane; intrinsic to plasma membrane;membrane; membrane part; neuron projection; plasma membrane; plasma membrane part;postsynaptic membrane; presynaptic membrane; proteinaceous extracellular matrix; synapse part
	F	actin binding; adenyl nucleotide binding; adenyl ribonucleotide binding; ATP binding; binding; cadherin binding;calcium channel activity; calcium ion binding; cation binding; cation channel activity; cell adhesion molecule binding; channel activity; cytoskeletal protein binding; diacylglycerol binding;extracellular-glutamate-gated ion channel activity; gated channel activity; glutamate receptor activity; GTPase regulator activity; guanyl-nucleotide exchange factor activity;ion binding; ion channel activity; ion transmembrane transporter activity;ionotropic glutamate receptor activity; kinase activity; ligand-gated channel activity;ligand-gated ion channel activity; metal ion binding; metal ion transmembrane transporter activity;molecular transducer activity; nucleoside binding; nucleoside-triphosphatase regulator activity;passive transmembrane transporter activity; phosphoric diester hydrolase activity;phosphotransferase activity, alcohol group as acceptor; protein kinase activity;protein tyrosine kinase activity; protein tyrosine phosphatase activity; purine nucleoside binding;Rho GTPase activator activity; signal transducer activity; transferase activity, transferring phosphorus-containing groups; transmembrane receptor protein kinase activity;transmembrane receptor protein tyrosine kinase activity
	P	cell adhesion; biological adhesion; homophilic cell adhesion; cell-cell adhesion; ion transport; metal ion transport
5’-UTR	C	membrane attack complex
	F	calcium-dependent protein binding
	P	none
3’-UTR	C	glycerol-3-phosphate dehydrogenase complex
	F & P	none
Promoter	C	synaptosome
	F	transmembrane receptor activity; receptor activity; G-protein coupled receptor activity; signal transducer activity;molecular transducer activity; olfactory; voltage-gated ion channel activity; voltage-gated channel activity
	P	G-protein coupled receptor protein signaling pathway; cell surface receptor linked signal transduction;sensory perception of chemical stimulus; inorganic; anion transport

### Gene expression profiling

In each epileptic sample, an average of 78044550.80±8806016.67 reads covering 7024009572.00±792541500.40 bp were sequenced, among which an average 83.71% of reads were uniquely mapped to reference genome, and 66.95% of reads were uniquely mapped to reference genes. In controls, an average of 79185195.80±9170582.72 reads covering 7126667622.00±825352445.10 bp were sequenced, among which an average 83.80% of reads were uniquely mapped to reference genome, and 65.99% of reads were uniquely mapped to reference genes. No significant difference was found between the two groups by T test (p<0.05 was considered statistically significant). (Supplementary Table S3).

A total of 21353 genes were sequenced, among which 17665 genes were expressed by all samples. The sequencing coverage of 65.01% and 65.45% genes was over 90% in epileptic samples and controls, respectively, indicating a good sequencing quality.

**Figure 2:**
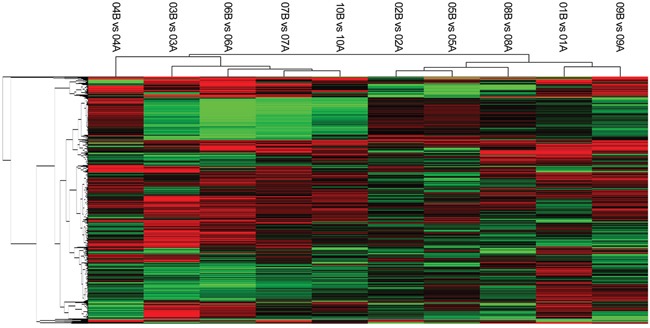
The gene expression signature of differentially expressed genes in ≥ 5 pairs of samples (purple: expression up; green: expression down; black: no difference). The hierarchical cluster showed a distinct expression signature with viariation between the paired samples.

Pairwise comparison identified 8850 differentially expressed genes (DEG), among which 885 genes were differentially expressed in ≥5 of the 10 pairs (Figure 2). Out of the 8850 DEGs, 246 were epilepsy-related genes according to NCBI Gene (http://www.ncbi.nlm.nih.gov/gene), and 34 epilepsy-related genes were differentially expressed in ≥5 of the 10 pairs (Table 3). However, only 3 out of the 65 genes differentially expressed in ≥8 pairs of the 10 pairs were epilepsy-related genes according to NCBI Gene (Table 4), which suggested that some new DEGs, including AZGP1, MMP19, DES, LGR6, SERPINA3, CX3CR1, DUSP5, EGR4, GPR37, etc. might be correlated with refractory epilepsy.

**Table 3:** Differentially expressed epilepsy-relate genes in ≥ 5 pairs of samples

Symbol	description	Epilepsy-Related Disorders
AQP1	aquaporin 1	refractory epilepsy/mesial temporal lobe sclerosis
ATF3	activating transcription factor 3	refractory mTLE
C3	complement component 3	TLE/FS/seizures following acute viral infection
CALB2	calbindin 2	TLE/FCD/LTP
CCR5	chemokine (C-C motif) receptor 5	refractory epilepsy/infantile-onset epilepsy/SUDEP/SE
EGR1	early growth response 1	IAE/focal epilepsy
EMP1	epithelial membrane protein 1	refractory epilepsy
GRIN2B	glutamate receptor, ionotropic, N-methyl D-aspartate 2B	West syndrome/FCD/TLE/anti-NMDAR encephalitis
HLA-DQA1	major histocompatibility complex, class II, DQ alpha 1	IGE/JME/absence epilepsy
HP	haptoglobin	IGE/familial epilepsy
IL1A	interleukin 1, alpha	TLE/FS
IL1B	interleukin 1, beta	epilepsy
IL1RN	interleukin 1 receptor antagonist	TLE
IL6	interleukin 6	TLE/FS/familial epilepsy/refractory epilepsy
CXCL8	chemokine (C-X-C motif) ligand 8	refractory epilepsy
IL18	interleukin 18	seizures in MS
ITGA2	integrin, alpha 2	refractory epilepsy
KCNA1	potassium channel, voltage gated shaker related subfamily A, member 1	SUDEP/TLE/partial epilepsy/Myokymia
NPY	neuropeptide Y	absence epilepsy/TLE/SE
OPRM1	opioid receptor, mu 1	tonic-clonic seizures/TLE/IGE/SE/IAE
PDYN	prodynorphin	TLE/FLTLE
RELN	reelin	TLE
PTGS2	prostaglandin-endoperoxide synthase 2	mTLE/absence seizures/poststroke seizures
SCN1B	sodium channel, voltage gated, type I beta subunit	Genetic GEFS+/Dravet Syndrome/convulsions with gastroenteritis/BPEI/LQTS/brugada syndrome
SCN5A	sodium channel, voltage gated, type V alpha subunit	Dravet syndrome/SUDEP/BFNS/LQTS/brugada syndrome
CCL2	chemokine (C-C motif) ligand 2	refractory epilepsy/SE
CCL4	chemokine (C-C motif) ligand 4	TLE
CDKL5	cyclin-dependent kinase-like 5	West syndrome/early-onset epileptic encephalopathies
CNTN2	contactin 2 (axonal)	PME/Autosomal recessive epilepsy/Autoimmune epilepsy
TNF	tumor necrosis factor	TLE/refractory epilepsy
TRPC4	transient receptor potential cation channel, subfamily C, member 4	generalized epilepsy with photosensitivity
CACNA1H	calcium channel, voltage-dependent, T type, alpha 1H subunit	IGE/CAE/generalized epilepsy syndromes
PLCB1	phospholipase C, beta 1 (phosphoinositide-specific)	early infantile epilepsy syndromes/malignant migrating partial seizures in infancy
ERMN	ermin, ERM-like protein	epileptic seizure/oligodendrocytes and epilepsy

mTLE: mesial temporal lobe epilepsy; TLE: temporal lobe epilepsy; FS: febrile seizures; FCD: focal cortical dysplasia; LTP: long-term potentiation; SUDEP: sudden unexpected death in epilepsy; SE: status epilepticus; IAE: idiopathic absence epilepsy; IGE: idiopathic generalized epilepsy; JME: juvenile myoclonic epilepsy; MS: multiple sclerosis; FLTLE: familial lateral temporal lobe epilepsy; GEFS+: generalized epilepsy with febrile seizure plus; BPEI: benign partial epilepsy in infancy; LQTS: long QT syndrome; BFNS: benign familial neonatal seizure; PME: progressive myoclonic epilepsy; CAE: childhood absence epilepsy.

**Table 4:** Differentially expressed genes in ≥8 pairs of the samples

Symbol	Description	Symbol	Description
SERPINA3	serpin peptidase inhibitor, clade A (alpha-1 antiproteinase, antitrypsin), member 3	SOCS3	suppressor of cytokine signaling 3
AQP1^a^	aquaporin 1 (Colton blood group)	CH25H	cholesterol 25-hydroxylase
AZGP1	alpha-2-glycoprotein 1, zinc-binding	CD163	CD163 molecule
BMP5	bone morphogenetic protein 5	CARTPT	CART prepropeptide
CHRM5	cholinergic receptor, muscarinic 5	KCNH4	potassium channel, voltage gated eag related subfamily H, member 4
CX3CR1	chemokine (C-X3-C motif) receptor 1	CDH19	cadherin 19, type 2
DES	desmin	BLNK	B-cell linker
DUSP5	dual specificity phosphatase 5	GCNT4	glucosaminyl (N-acetyl) transferase 4, core 2
EGR4	early growth response 4	TNFRSF12A	tumor necrosis factor receptor superfamily, member 12A
FCGR3A	Fc fragment of IgG, low affinity IIIa, receptor (CD16a)	CD244	CD244 molecule, natural killer cell receptor 2B4
FOS	FBJ murine osteosarcoma viral oncogene homolog	LGR6	leucine-rich repeat containing G protein-coupled receptor 6
FOSB	FBJ murine osteosarcoma viral oncogene homolog B	STRA6	stimulated by retinoic acid 6
GPR37	G protein-coupled receptor 37 (endothelin receptor type B-like)	SH3TC2	SH3 domain and tetratricopeptide repeats 2
GRIN2B^a^	glutamate receptor, ionotropic, N-methyl D-aspartate 2B	DCSTAMP	dendrocyte expressed seven transmembrane protein
HBA2	hemoglobin, alpha 2	CNDP1	carnosine dipeptidase 1 (metallopeptidase M20 family)
HBB	hemoglobin, beta	PNMA6A	paraneoplastic Ma antigen family member 6A
SERPIND1	serpin peptidase inhibitor, clade D (heparin cofactor), member 1	SLC5A11	solute carrier family 5 (sodium/inositol cotransporter), member 11
HDC	histidine decarboxylase	KIF19	kinesin family member 19
HLA-DRB5	major histocompatibility complex, class II, DR beta 5	FREM3	FRAS1 related extracellular matrix 3
HSD11B1	hydroxysteroid (11-beta) dehydrogenase 1	DLGAP1-AS3	DLGAP1 antisense RNA 3
HTR3A	5-hydroxytryptamine (serotonin) receptor 3A, ionotropic	C20orf166-AS1	C20orf166 antisense RNA 1
TNC	tenascin C	NPAS4	neuronal PAS domain protein 4
IL6^a^	interleukin 6	HMGA1P7	high mobility group AT-hook 1 pseudogene 7
ITK	IL2-inducible T-cell kinase	C11orf96	chromosome 11 open reading frame 96
LRP2	low density lipoprotein receptor-related protein 2	TMEM233	transmembrane protein 233
MBP	myelin basic protein	TMEM215	transmembrane protein 215
MC4R	melanocortin 4 receptor	CTXN3	cortexin 3
MMP19	matrix metallopeptidase 19	LOC643711	platelet-activating factor acetylhydrolase 1b, catalytic subunit 2 (30kDa) pseudogene
NPPA	natriuretic peptide A	LOC100130331	POTE ankyrin domain family, member F pseudogene
VCAM1	vascular cell adhesion molecule 1	CD24	CD24 molecule
ZFP36	ZFP36 ring finger protein	BRE-AS1	BRE antisense RNA 1
NR4A3	nuclear receptor subfamily 4, group A, member 3	LINC00507	long intergenic non-protein coding RNA 507
BAIAP3	BAI1-associated protein 3		

a: Epilepsy-related genes according to NCBI Gene (http://www.ncbi.nlm.nih.gov/gene).

Pairwise comparison presented a total of 395 GO terms significantly enriched in epileptic samples compared to controls (Component 68, Function 70, Process 257). 77 GO terms were significantly enriched in ≥5 epileptic samples compared to controls, including 12 component terms, 23 function terms and 42 process terms (Table 5). The GO enrichment analysis of gene expression revealed that the biological functions of DEGs are mainly correlated with channel activity, transporter activity, and receptor activity. Furthermore, the biological processes mainly targeted by DEGs were immune system, biological regulation, response to stimulus, signaling, development, and behavior.

**Table 5:** Enriched GO terms and KEGG pathways in epileptic sample

Term	Genes	Term	Genes
GO Component			
GO:0071944-Cell periphery	516	GO:0016020-Membrane	2319
GO:0009986-Cell surface	48	GO:0044425-Membrane part	1912
GO:0005576-Extracellular region	333	GO:0016021-Integral to membrane	509
GO:0044421-Extracellular region part	326	GO:0031224-Intrinsic to membrane	1654
GO:0031012-Extracellular matrix	114	GO:0005886-Plasma membrane	511
GO:0005578-Protenaceous extracellular matrix	44	GO:0044459-Plasma membrane part	488
GO Function			
GO:0015267-Channel activity	172	GO:0001653-Peptide receptor activity	39
GO:0005261-Cation channel activity	113	GO:0008528-Peptide receptor activity, G-protein coupled	36
GO:0005216-Ion channel activity	167	GO:0060089-Molecular transducer activity	586
GO:0022838-Substrate-specific channel activity	167	GO:0004871-Signal transducer activity	506
GO:0005215-Transporter activity	402	GO:0005515-Protein binding	1337
GO:0022857-Transmembrane transporter activity	241	GO:0005102-Receptor binding	286
GO:0015075-Ion transmembrane transporter activity	276	GO:0001871-Pattern binding	59
GO:0022803-Passive transmembrane transporter activity	173	GO:0042277-Peptide binding	50
GO:0022892-Substrate-specific transporter activity	367	GO:0030246-Carbohydrate binding	120
GO:0022891-Substrate-specific transmembrane transporter activity	220	GO:0030247-Polysaccharide binding	57
GO:0004872-Receptor activity	347	GO:0005539-Glycosaminoglycan binding	52
GO:0004888-Transmembrane receptor activity	225		
GO Process			
GO:0001775-Cell activation	421	GO:0009605-Response to external stimulus	42
GO:0007154-Cell communication	120	GO:0009617-Response to bacterium	201
GO:0030154-Cell differentiation	1390	GO:0009991-Response to extracellular stimulus	287
GO:0002376-Immune system process	449	GO:0031667-Response to nutrient levels	79
GO:0032501-Multicellular organismal process	604	GO:0009611-Response to wounding	168
GO:0032501-Biological regulation	160	GO:0023052-Signaling	75
GO:0050789-Regulation of biological process	1153	GO:0019932-Second-messenger-mediated signaling	308
GO:0048518-Positive regulation of biological process	146	GO:0023033-Signaling pathway	96
GO:0065008-Regulation of biological quality	326	GO:0007166-Cell surface receptor linked signaling pathway	1022
GO:0050793-Regulation of developmental process	283	GO:0007186-G-protein coupled receptor protein signaling pathway	434
GO:0002682-Regulation of immune system process	898	GO:0009653-Anatomical structrue morphogenesis	67
GO:0051239-Regulation of multicellular organismal process	1211	GO:0048856-Anatomical structure development	89
GO:0050896-Response to stimulus	496	GO:0007275-Multicellular organismal development	239
GO:0042221-Response to chemical stimulus	527	GO:0032502-Developmental process	614
GO:0010033-Response to organic substance	1625	GO:0048869-Cellular developmental process	332
GO:0006950-Response to stress	669	GO:0048731-System development	1342
GO:0006952-Defense response	259	GO:0048513-Organ development	906
GO:0009719-Response to endogenous stimulus	201	GO:0009888-Tissue development	824
GO:0009725-Response to hormone stimulus	350	GO:0007610-Behavior	988
GO:0048545-Response to steroid hormone stimulus	59	GO:0006811-Ion transport	222
GO:0031960-Response to corticosteroid stimulus	615	GO:0048878-Chemical homeostasis	182
KEGG Pathway			
ko05143-African trypanosomiasis	36	ko04640-Hematopoietic cell lineage	110
ko05330-Allograft rejection	38	ko04672-Intestinal immune network for IgA production	37
ko05146-Amoebiasis	120	ko05140-Leishmaniasis	87
ko05310-Asthma	24	ko04670-Leukocyte transendothelial migration	151
ko05320-Autoimmune thyroid disease	43	ko05144-Malaria	47
ko04662-B cell receptor signaling pathway	92	ko04650-Natural killer cell mediated cytotoxicity	109
ko04020-Calcium signaling pathway	177	ko04080-Neuroactive ligand-receptor interaction	244
ko04514-Cell adhesion molecules	137	ko04380-Osteoclast differentiation	127
ko04062-Chemokine signaling pathway	151	ko04145-Phagosome	197
ko04610-Complement and coagulation cascades	113	ko05020-Prion diseases	54
ko04060-Cytokine-cytokine receptor interaction	171	ko05323-Rheumatoid arthritis	78
ko04512-ECM-receptor interaction	155	ko05150-Staphylococcus aureus infection	85
ko04666-Fc gamma R-mediated phagocytosis	116	ko04940-Type I diabetes mellitus	45
ko05332-Graft-versus-host disease	43		

In Kyoto Encyclopedia of Genes and Genome (KEGG) pathway enrichment analysis, 75 pathways were significantly enriched in epileptic samples compared to controls, while 27 pathways were significantly enriched in ≥5 epileptic samples compared to controls (Table 5). DEGs mainly participate in calcium signaling pathway, neuroactive ligand-receptor interaction, and pathways involved in inflammation, immune response, and autoimmune diseases.

### DNA methylation and gene expression

**Figure 3:**
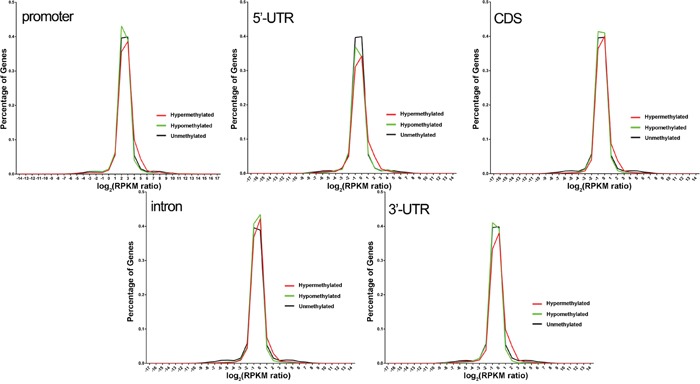
The distribution of hyper-, hypo- and unmethylated gene expression levels in different gene elements No significant relationship was found between DNA methylation and gene expression in all the 5 gene elements, and the percentage of hyper-methylated genes with log2(RPKM ratio) approximately ranging from 1 to 3 were higher than that of hypo-methylated and unmethylated genes.

The distribution of hyper-, hypo- and unmethylated gene expression levels in different gene elements were presented in Figure 3. The trend of gene expression of the three groups were similar, and in all the 5 elements, the percentage of hyper-methylated genes with log_2_(RPKM ratio) (RPKM ratio = RPKM of epileptic sample/RPKM of control) approximately ranging from 1 to 3 were higher than that of hypo-methylated and unmethylated genes. Generally, no significant relationship in modulation was found between DNA methylation and gene expression.

## DISCUSSION

It is the first genome-wide report on DNA methylation and gene expression in refractory epilepsy patients. 62 differentially expressed genes such as MMP19, AZGP1, DES, and LGR6 were discovered to be correlated with refractory epilepsy, and diverse distribution of differentially methylated genes was found in X chromosome instead of in Y chromosome.

Although many genes were observed differentially methylated or expressed, the general distribution of DNA methylation reads, DNA methylation peaks and mRNA sequencing reads were similar between refractory epileptic samples and controls, which indicate no significant difference in global DNA methylation and global gene expression between the two groups. Inconsistent with previous report which identified decreased DNA methylation in Y chromosome of TLE patients [18], our study presented no difference in DNA methylation in Y chromosome between refractory epileptic samples and controls. It is noteworthy that 4 out of the 5 male epileptic samples in our study were frontal lobe epilepsy, suggesting a possible difference in DNA methylation in Y chromosome between frontal lobe epilepsy and TLE. On the contrary to a previous research which found no change of DNA methylation in X chromosome in rat models of epilepsy [17], we identified diverse DMR distribution on X chromosome in all patients. This difference may be attributed to the much more complicated environmental factors involved by human beings or the difference between species.

In pairwise comparison of gene expression analysis, we identified distinct gene expression signatures. 34 DEGs are correlated with epilepsy or seizure and 14 of these genes are associated with refractory epilepsy, such as AQP1 [21], CCR5 [22], EMP1 [23], CXCL8 [24], ITGA2 [25], and CCL2 [26]. For the first time, 62 DEGs differentially expressed in ≥8 pairs of samples were found related to epilepsy/seizure in our study. These newly-identified refractory epilepsy-related genes may possibly reveal new mechanisms of refractory epilepsy. In all the DEGs, only MMP19 and AZGP1 were differentially expressed in all the 10 pairs. Compared to the controls, 9 of the 10 epileptic samples showed increased expression of MMP19, while 1 epileptic sample showed decreased expression. MMP19 and other matrix metalloproteinases can cleave and remodel the extracellular matrix, including tenascin and laminin, and thus influence synapse formation and remodeling, N-methyl-D-aspartate receptor activity, learning and memory, and hippocampal long-term potentiation [27]. Inhibition of MMP19 and other matrix metalloproteinases may prevent development of epilepsy at the early stage of epileptogenesis [28]. Meanwhile, we found the expression of AZGP1 were decreased in 8 of the 10 epileptic samples, and increased in 2 of the 10 epileptic samples when compared to controls. AZGP1 encodes Zinc-a2-glycoprotein, which is an adipokine participates in lipid mobilization, lipolytic effect, and immune response [29, 30]. Moreover, both DES in 9 pairs and LGR6 in 8 pairs showed consistently increased expression in epileptic samples. To the best of our knowledge, it is the first time that MMP19, AZGP1, DES, and LGR6 were reported to be correlated with refractory epilepsy.

Significant enrichment of DMGs in GO terms of binding, transport, and enzymatic activity were found, which is consistent with previous studies [18]. Interestingly, most of the DMGs were differentially methylated in CDS and intron, while previous research showed differential methylation in all the gene elements in rat models of chronic epilepsy induced by pilocarpine [17]. These findings indicate DNA methylation in CDS and intron may play critical roles in refractory epilepsy besides promoter methylation which has been a very popular target in research on epilepsy [18, 31]. The GO enrichment analysis of gene expression revealed a trend similar to a previous report [17] that the DEGs are mainly correlated with biological functions such as protein binding, receptor binding, channel activity, transporter activity, and receptor activity, as well as being involved in biological processes such as immune system, biological regulation, response to stimulus, signaling, development, and behavior.

The change of DNA methylation in this study is not exactly corresponded with alteration of gene expression. Kobow and his colleagues found that DNA methylation in promoter, exon and intron were inversely correlated with gene expression in rat models of chronic epilepsy induced by pilocarpine [17], but our study found that most of the hyper- and hypo-methylated genes were not differentially expressed in epilepsy patients, and the percentage of hyper-methylated genes with log2(RPKM ratio) ranging from 1 to 3 were higher than that of hypo-methylated and unmethylated genes, which indicate a complicated modulation between DNA methylation and gene expression in refractory epilepsy in human beings.

In KEGG analysis of gene expression, DEGs significantly enriched in calcium signaling pathway, neuroactive ligand-receptor interaction, and pathways participating in inflammation, immune response, autoimmune diseases. Calcium signaling pathway has been increasingly recognized as a vital factor in epileptogenesis and the excess synchronization, and hyperexcitability of neurons for seizures can be linked to various calcium signaling pathways [32]. The aberrantly neuroactive ligand-receptor interaction can enhance the susceptibility to epileptic seizures [33, 34]. It may explain partially that the drugs regulating the function of calcium signaling pathway and neuroactive ligand-receptor interaction are able to alleviate the seizure frequency [35]. The roles of immune response and inflammation in epilepsy have been recognized in previous studies [36]. Autoimmune epilepsy frequently present drug-resistance which can be controlled by immunosuppressive and immunomodulatory therapies [36, 37]. Consistent with Lukic and his colleagues’ study, we also identified that prion disease was significantly targeted by DEGs which indicate both refractory epilepsy and prion diseases may share some common pathway [38].

## MATERIALS AND METHODS

### Study approval

The research protocol was approved by the Ethics Committees of the Second Affiliated Hospital of Chongqing Medical University. All procedures performed in studies involving human participants were in accordance with the ethical standards of the institutional and/or national research committee and with the 1964 Helsinki declaration. Written informed consent was obtained from all individual participants included in the study or their proxies.

### Patients and tissues preparation

Resected brain tissues were retrospectively but consecutively collected from 10 patients with refractory epilepsy and 10 patients with post-traumatic intracranial hypertension who underwent surgical treatment since 2008 to 2014. Patients with refractory epilepsy were diagnosed following the definition of ILAE [39]. Briefly, all patients were resistant to maximum doses of at least three anti-epileptic drugs (AED), and evaluated by detailed history, neurological examination, neuropsychological test and neuroimaging data. For presurgical evaluation and epileptogenic zones identification, a combined assessment of ictal simiology, brain magnetic resonance imaging, video-electroencephalography, sphenoidal electrode monitoring and intracerebral electroencephalography and intraoperative electrocorticography were applied. After evaluation, standard en bloc resection was performed. No refractory epilepsy patient received adjustment of AEDs during the 2 months before surgery. Brain tissues as control from the 10 post-trauma intracranial hypertension patients were identified normal by neuropathologist. These patients had no history of epilepsy or exposure to AEDs. All the resected brain tissues were immediately frozen in liquid nitrogen and then stored at -80°C.

The 10 epileptic samples and 10 controls were paired, the difference in genome-wide DNA methylation and gene expression between the paired samples were analyzed using MeDIP-seq and mRNA-seq.

### DNA methylation profiling

Genomic DNA was extracted using QIAamp DNA Micro Kit (Qiagen, Hilden, Germany) according to the manufacturer's instruction. Extracted DNA was fragmented to a size of 100-500 bp by sonication (Bioruptor NGS, Digenode, Liege, Belgium), and subjected to DNA-end repair, 3’-dA overhang and ligation of sequencing adaptors according to manufacturer's instruction (Paired-End DNA Sample Prep kit, Illumina, San Diego, USA) and denatured to single-stranded. Then the methylated DNA was immunoprecipitated by 5mc antibody (Magnetic Methylated DNA Immunoprecipitation kit, Diagenod, Liege, Belgium). After Real-time Quantitative polymerase chain reaction (PCR) (TaqMan Probe, Applied Biosystems, Thermo Fisher Scientific, Waltham, USA) validation and quality control of sample library (Agilent 2100 BioAnalyzer, Agilent, Santa Clara, USA), electrophoretically selected DNA fragments sizing from 200-300 bp were subjected to high-throughput sequencing (Illumina HiSeqTM 2000, Illumina, San Diego, USA). Sequencing strategy was Single-end 50 bp, and reads size was 49 bp.

Filtered MeDIP-Seq data (Adapters, reads containing more than 10% bases undetermined, and low quality reads were removed. Low quality read means the quality values (Q) of more than 50% bases in this read were ≤20, Q=-10lg(rate of sequencing error)) was mapped to reference genome using SOAP software, version 2.21 (Website: http://soap.genomics.org.cn)[40], only unique alignments with no more than 2 mismatches were included for further analysis. The reference genome data and the data used for annotation of all aligned genes were from UCSC Genome Bioinformatics Download (Reference genome: http://hgdownload.cse.ucsc.edu/goldenPath/hg19/chromosomes/chr*.fa.gz. Reference genes: http://hgdownload.cse.ucsc.edu/goldenPath/hg19/bigZips/chromOut.tar.gz).

To describe the distribution of MeDIP-Seq data on genome, the following items were calculated: 1) Genome coverage distribution across sequencing depth; 2) Distribution of CpG, CHG and CHH sites varies with sequencing depth; 3) Reads distribution in genome regions with different CpG density; 4) Distribution of reads in different gene elements, including CpG islands, promoters, 5’-Untranslated regions (UTR), CDS, introns, 3’-UTR, repeat regions and each class of repetitive elements (Repeat dataset is obtained from RepeatMasker (Transposons) and Tandem Repeats Finder (Tandom repeats), and is available at:http://hgdownload.cse.ucsc.edu/goldenPath/hg19/bigZips/chromOut.tar.gz); 5) Distribution of MeDIP-Seq reads around CpG island and gene body.

Whole genome scanning of enrichment region of methylation/Peak was based on a defined analysis model, MACS 1.4.0 (Website:http://liulab.dfci.harvard.edu/MACS/) with default parameters [41]. The following items were calculated: 1) Distribution of peaks with different length; 2) Distribution of peaks with different CpG density; 3) Number and coverage of peaks in gene elements (promoter, 5’-UTR, CDS, intron, and 3’-UTR).

DMGs based on peak of all the paired samples were analyzed. Briefly, peaks of the paired two samples were merged as candidate DMRs. For each candidate DMR, the number of reads of each sample was calculated and tested to get true DMRs. The downtrend DMRs indicate that the number of reads of the control sample was larger than the epileptic sample and the uptrend DMRs indicate the opposite. DMGs were defined as the genes overlapping DMRs. Genes with an element merely overlap uptrend DMRs were considered hyper-methylated with such an element, genes with an element merely overlap downtrend DMRs were considered hypo-methylated with such an element.

To clarify the biological functions of DMGs, GO enrichment analysis was performed. Briefly, DMGs were mapped to GO terms in reference database (http://www.geneontology.org) and gene numbers for every term were calculated and tested to identify the significantly enriched GO terms.

### Gene expression profiling

RNA was extracted using Trizol method. After DNase I treatment, mRNA was isolated with magnetic beads with Oligo (dT) and fragmented. Then cDNA was synthesized using the mRNA fragments as templates. The synthesized cDNA fragments were purified and subjected to end reparation, single nucleotide adenine addition and adapter connection. cDNA fragments suitable for PCR amplification were selected with electrophoresis. Quality control of sample library was performed using Agilent 2100 Bioanaylzer (Agilent, Santa Clara, USA) and Applied Biosystems StepOnePlus Real-Time PCR System (Applied Biosystems, Thermo Fisher Scientific, Waltham, USA). The library was sequenced using Illumina HiSeqTM 2000 (Illumina, San Diego, USA).

After sequencing quality control, the mRNA-Seq data was mapped to reference genome and reference genes using SOAP software, version 2.21 (Website:http://soap.genomics.org.cn) [40]. Then the distribution of reads on reference genome and genes was calculated and coverage analysis was performed. After alignment quality control, the DEGs were selected. And for further analysis, expression pattern analysis of DEGs were also performed.

To clarify the biological functions of DEGs, GO enrichment analysis of DEGs was performed as described above. To further clarify the biological functions of DEGs, KEGG pathway analysis was performed using the same calculating formula as GO enrichment analysis with database available at http://www.kegg.jp/kegg/.

### Correlation analysis of DNA methylation and gene expression

The distribution of hyper-, hypo- and unmethylated gene expression levels in different gene elements were calculated to analyze the relationship between DNA methylation and gene expression as previously described [42].

### Statistics and analysis

To identify true DMRs, the numbers of reads were calculated and assessed using chi-square statistics and False discovery rate (FDR) statistics (p≤0.01, and the difference of read numbers should be more than twice). To identify significantly enriched GO terms and KEGG pathways, gene numbers for every term or pathway were calculated and then assessed using hypergeometric test, p-value of hypergeometric test was corrected using Bonferroni Correction [43]. GO terms with corrected p-value ≤0.01, and KEGG pathways with corrected p-value ≤0.05 were considered significantly enriched. In selection of DEGs, the gene expression level was calculated using RPKM method [44], and DEGs were selected as previously described [45]. The adjusted p-value was calculated using Benjamini, Yekutieli. 2001 FDR method [46] and DEGs was defined as genes with FDR≤0.001 and the RPKM difference between the paired samples should be more than twice. Hierarchical cluster was performed to analyze the expression pattern of DEGs using Cluster [47] and presented using Java Treeview [48]. The DEGs were clustered by Euclidean distance.

### Abbreviations

CDS=coding sequences; DMG=differentially methylated genes; DMR=Differentially Methylated Regions; DEG=differentially expressed genes; GO=gene ontology; ILAE=International League Against Epilepsy; KEGG=Kyoto Encyclopedia of Genes and Genome; MeDIP-Seq=methylated DNA immunoprecipitation linked with sequencing; mRNA-Seq=mRNA sequencing; PCR=polymerase chain reaction; RPKM=Reads Per Kilobases per Millionreads; TLE=temporal lobe epilepsy; UTR=Untranslated regions.

## SUPPLEMENTARY TABLES


