# Supplementary material for: New differentially expressed genes and differential DNA methylation underlying refractory epilepsy
Source: Oncotarget. 2016 Nov 26;7(52):87402–16. doi: 10.18632/oncotarget.13642 (PMC5349997; doi:10.18632/oncotarget.13642)
Supplement: Supplementary file 1 [file oncotarget-07-87402-s001.pdf]

New differentially expressed genes and differential DNA methylation underlying refractory epilepsy

SUPPLEMENTARY TABLES

Supplementary Table S1: Brain regions of samples in patients

Sample	Age	Sex	Region	Onset	Duration	Event
01A	21	m	RF	1y3mon	20y	None
02A	23	m	LF	1y	22y	None
03A	18	m	LF	3y	15y	None
04A	19	m	LP+LO	15y	4y	Schizencephaly
05A	21	m	LF	3y	18y	None
06A	19	f	LF	4y	15y	None
07A	17	f	RT	16y	1y	None
08A	16	f	LT	7y	9y	None
09A	2	f	LF	0y8mon	1y4mon	Viral encephalitis
10A	15	f	RF	14y	1y	None

m: male; f: female; RF: right frontal lobep; LF: left frontal lobe; LO: left occipital lobe; RT: right temporal lobe; LT: left temporal lobe; y: year; mon: month.

Supplementary Table S2: Detailed data of methylation reads and peaks

Sample	Total Reads	Mapped Reads	Mapping Rate	Unique Mapped Reads	Unique Mapping Rate	Total Peaks	Peak Mean Length	Peak	Peak
01A[a]	81,632,654	76,963,255	94.28	58,514,607	71.68	68,653	1350.03	92,683,399	2.95
01B[b]	81,632,654	76,610,184	93.85	58,582,820	71.76	116,296	1385.62	161,142,114	5.14
02A	81,632,654	76,486,214	93.7	58,402,320	71.54	108,656	1308.16	142,139,935	4.53
02B	81,632,654	77,461,391	94.89	61,356,385	75.16	90,698	1434.38	130,095,769	4.15
03A	81,632,654	76,970,014	94.29	59,105,761	72.4	111,810	1334.87	149,251,752	4.76
03B	81,632,654	75,673,881	92.7	54,559,630	66.84	146,733	1260.35	184,935,147	5.89
04A	81,632,654	76,782,253	94.06	58,806,391	72.04	113,738	1361.46	154,850,004	4.94
04B	81,632,654	75,848,662	92.91	59,457,341	72.84	76,717	1468.85	112,686,009	3.59
05A	81,632,654	76,541,735	93.76	59,970,841	73.46	100,378	1346.18	135,126,976	4.31
05B	81,632,654	76,168,081	93.31	56,635,996	69.38	82,841	1382.17	114,500,711	3.65
06A	81,632,654	75,265,531	92.2	55,550,422	68.05	129,027	1324.93	170,951,682	5.45
06B	81,632,654	75,800,909	92.86	59,366,061	72.72	104,823	1387.47	145,439,169	4.64
07A	81,632,654	75,708,572	92.74	58,084,418	71.15	115,136	1321.44	152,145,268	4.85
07B	81,632,654	76,845,362	94.14	61,086,322	74.83	94,911	1370.84	130,107,854	4.15
08A	81,632,654	75,687,929	92.72	58,408,516	71.55	126,870	1351.64	171,482,561	5.47
08B	81,632,654	73,907,624	90.54	49,565,816	60.72	152,299	1144.65	174,329,761	5.56
09A	81,632,654	75,278,972	92.22	53,684,776	65.76	152,139	1249.49	190,095,571	6.06
09B	81,632,654	77,143,624	94.5	60,349,095	73.93	114,896	1366.86	157,046,897	5.01
10A	81,632,654	76,702,011	93.96	60,672,708	74.32	124,874	1355.24	169,234,750	5.39
10B	81,632,654	76,072,199	93.19	56,734,074	69.5	129,988	1313.79	170,777,125	5.44

a: The letter "A" following sample number (for example: 01A) indicates epileptic sample;
b: The letter "B" following sample number (for example: 01B) indicates control, and epileptic samples and controls with the same sample number were taken into pairwise comparison (for example: 01A vs. 01B).

Supplementary Table S3: Detailed data of mRNA-seq

Sample	Clean reads	Genome map Rate (%)	Gene map Rate (%)	Expressed Gene	Total Reads	Total BasePairs	Total Mapped Reads to Genome	Unique Mapped Read to Genome	Total Mapped Reads to Genes	Unique Mapped Reads to Genes
01A	97045478	87.50	69.08	19760	97045478	8734093020	84911012	81158468	67042488	64883639
01B	76426072	87.16	66.55	19525	76426072	6878346480	66612085	63959980	50862550	49586550
02A	67036536	87.14	70.23	19307	67036536	6033288240	58414527	56092288	47079916	45855455
02B	71179770	87.67	70.61	19408	71179770	6406179300	62400479	59609475	50258323	48875774
03A	85616198	87.64	71.65	19542	85616198	7705457820	75032963	71801918	61342653	59720613
03B	83436668	87.46	70.68	19312	83436668	7509300120	72972320	70037989	58977153	57442283
04A	71400528	87.42	65.75	19531	71400528	6426047520	62417677	59862464	46944747	45681925
04B	66810912	87.17	66.99	19390	66810912	6012982080	58240787	55874950	44754159	43562720
05A	70686548	87.21	66.82	19379	70686548	6361789320	61645240	59145546	47233078	45984877
05B	86280644	87.74	68.23	19749	86280644	7765257960	75705119	72671202	58868878	57295502
06A	71283184	86.50	68.97	19415	71283184	6415486560	61661708	59340207	49167327	47997630
06B	90615348	87.45	64.69	19533	90615348	8155381320	79246704	75964440	58621129	56926476
07A	79758516	87.09	70.14	19497	79758516	7178266440	69463682	66576621	55941869	54473654
07B	78097952	87.59	68.62	19337	78097952	7028815680	68403315	65464826	53591566	52132378
08A	77022412	88.21	68.11	19406	77022412	6932017080	67944139	65202109	52460165	51056338
08B	81519066	86.78	71.91	19391	81519066	7336715940	70742023	67897396	58623772	57121102
09A	79956310	86.88	69.10	19577	79956310	7196067900	69465042	66834685	55246280	53907742
09B	65975922	87.54	66.96	19422	65975922	5937832980	57753563	55367712	44178044	42983858
10A	80639798	86.73	67.28	19597	80639798	7257581820	69938224	67286805	54253679	52949995
10B	91509604	87.74	63.73	19657	91509604	8235864360	80290519	76748079	58316623	56594286

a: The letter "A" following sample number (for example: 01A) indicates epileptic sample;
b: The letter "B" following sample number (for example: 01B) indicates control, and epileptic samples and controls with the same sample number were taken into pairwise comparison (for example: 01A vs. 01B).
